# Dichloridobis(5,7-dichloro­quinolin-8-olato-κ^2^ *N*,*O*)tin(IV)

**DOI:** 10.1107/S1600536809004371

**Published:** 2009-02-13

**Authors:** Yousef Fazaeli, Ezzatollah Najafi, Mostafa M. Amini, Seik Weng Ng

**Affiliations:** a Department of Chemistry, General Campus, Shahid Beheshti University, Tehran 1983963113, Iran; b Department of Chemistry, University of Malaya, 50603 Kuala Lumpur, Malaysia

## Abstract

The Sn^IV^ atom in the title compound, [Sn(C_9_H_4_Cl_2_NO)_2_Cl_2_], is chelated by the substituted quinolin-8-olate anions in a distorted octa­hedral geometry. The N-donor atoms are in a *cis* alignment as are the Cl atoms; the O atoms are *trans* to each other.

## Related literature

For the structure of dichloridobis(quinolin-8-olato)tin(IV), which shows a very similar coordination geometry, see: Archer *et al.* (1987 ▶).
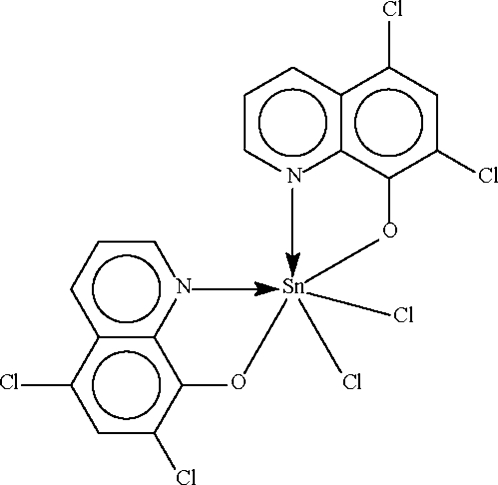


## Experimental

### 

#### Crystal data

[Sn(C_9_H_4_Cl_2_NO)_2_Cl_2_]
*M* *_r_* = 615.65
Monoclinic, 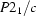

*a* = 15.2459 (2) Å
*b* = 8.9262 (1) Å
*c* = 15.8541 (2) Å
β = 110.381 (1)°
*V* = 2022.48 (4) Å^3^
*Z* = 4
Mo *K*α radiation
μ = 2.08 mm^−1^
*T* = 100 (2) K
0.28 × 0.22 × 0.18 mm

#### Data collection

Bruker SMART APEX diffractometer
Absorption correction: multi-scan (*SADABS*; Sheldrick, 1996 ▶) *T*_min_ = 0.594, *T*_max_ = 0.706
18582 measured reflections
4651 independent reflections
4218 reflections with *I* > 2σ(*I*)
*R*_int_ = 0.021

#### Refinement

*R*[*F* ^2^ > 2σ(*F* ^2^)] = 0.026
*wR*(*F* ^2^) = 0.079
*S* = 1.06
4651 reflections
262 parameters
H-atom parameters constrained
Δρ_max_ = 0.59 e Å^−3^
Δρ_min_ = −1.25 e Å^−3^

### 

Data collection: *APEX2* (Bruker, 2008 ▶); cell refinement: *SAINT* (Bruker, 2008 ▶); data reduction: *SAINT*; program(s) used to solve structure: *SHELXS97* (Sheldrick, 2008 ▶); program(s) used to refine structure: *SHELXL97* (Sheldrick, 2008 ▶); molecular graphics: *X-SEED* (Barbour, 2001 ▶); software used to prepare material for publication: *publCIF* (Westrip, 2009 ▶).

## Supplementary Material

Crystal structure: contains datablocks global, I. DOI: 10.1107/S1600536809004371/bt2866sup1.cif

Structure factors: contains datablocks I. DOI: 10.1107/S1600536809004371/bt2866Isup2.hkl

Additional supplementary materials:  crystallographic information; 3D view; checkCIF report

## Figures and Tables

**Fig. 1.**
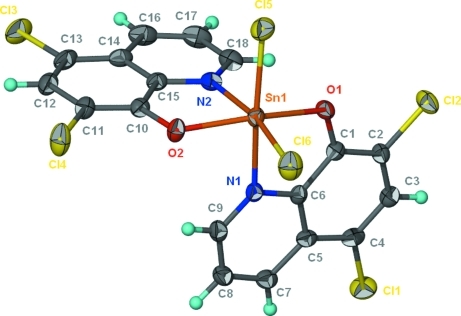
Anisotropic displacement ellipsoid plot (Barbour, 2001) of SnCl2(C9H4Cl2NO)2; ellipsoids are drawn at the 70% probability level and H atoms of arbitrary radius.

## supplementary crystallographic information

### Experimental

5,7-Dichloro-8-hydroxyquinoline (1 mmol, 0.21 g) was added to a solution of stannous chloride (1 mmol, 0.23) in DMSO (20 ml). The clear solution was set aside for several days to yield yellow crystals. These crystals are stable when heated at 573 K.

### Refinement

H-atoms were placed in calculated positions (C—H 0.93 Å) and were included in the refinement in the riding model approximation, with *U*(H) set to 1.2*U*(C). The final difference Fourier map had a large peak/deep hole at about 1 Å from the Sn atom.

### Crystal data

**Table:**

[Sn(C_9_H_4_Cl_2_NO)_2_Cl_2_]	*F*(000) = 1192
*M**_r_* = 615.65	*D*_x_ = 2.022 Mg m^−^^3^
Monoclinic, *P*2_1_/*c*	Mo *K*α radiation, λ = 0.71073 Å
Hall symbol: -P 2ybc	Cell parameters from 9940 reflections
*a* = 15.2459 (2) Å	θ = 2.6–28.3°
*b* = 8.9262 (1) Å	µ = 2.08 mm^−^^1^
*c* = 15.8541 (2) Å	*T* = 100 K
β = 110.381 (1)°	Polyhedron, yellow
*V* = 2022.48 (4) Å^3^	0.28 × 0.22 × 0.18 mm
*Z* = 4	

### Data collection

**Table:**

Bruker SMART APEX diffractometer	4651 independent reflections
Radiation source: fine-focus sealed tube	4218 reflections with *I* > 2σ(*I*)
graphite	*R*_int_ = 0.021
ω scans	θ_max_ = 27.5°, θ_min_ = 1.4°
Absorption correction: multi-scan (*SADABS*; Sheldrick, 1996)	*h* = −19→19
*T*_min_ = 0.594, *T*_max_ = 0.706	*k* = −11→11
18582 measured reflections	*l* = −20→20

### Refinement

**Table:**

Refinement on *F*^2^	Primary atom site location: structure-invariant direct methods
Least-squares matrix: full	Secondary atom site location: difference Fourier map
*R*[*F*^2^ > 2σ(*F*^2^)] = 0.026	Hydrogen site location: inferred from neighbouring sites
*wR*(*F*^2^) = 0.079	H-atom parameters constrained
*S* = 1.06	*w* = 1/[σ^2^(*F*_o_^2^) + (0.0394*P*)^2^ + 4.6706*P*] where *P* = (*F*_o_^2^ + 2*F*_c_^2^)/3
4651 reflections	(Δ/σ)_max_ = 0.001
262 parameters	Δρ_max_ = 0.59 e Å^−^^3^
0 restraints	Δρ_min_ = −1.25 e Å^−^^3^

### Fractional atomic coordinates and isotropic or equivalent isotropic displacement parameters (Å^2^)

**Table:**

	*x*	*y*	*z*	*U*_iso_*/*U*_eq_	
Sn1	0.778941 (13)	0.92337 (2)	0.344613 (13)	0.01736 (7)	
Cl1	0.93987 (6)	0.32043 (9)	0.64058 (5)	0.02934 (17)	
Cl2	0.84159 (6)	0.38698 (8)	0.28352 (5)	0.02702 (17)	
Cl3	0.34831 (5)	1.07293 (11)	0.39582 (6)	0.0343 (2)	
Cl4	0.66222 (6)	1.38854 (9)	0.45378 (7)	0.03409 (19)	
Cl5	0.70590 (6)	0.98399 (10)	0.19137 (5)	0.02889 (17)	
Cl6	0.92679 (6)	1.01709 (10)	0.36724 (6)	0.03319 (19)	
O1	0.80174 (15)	0.7039 (2)	0.31888 (13)	0.0191 (4)	
O2	0.73412 (14)	1.1077 (2)	0.39671 (15)	0.0205 (4)	
N1	0.83134 (16)	0.8252 (3)	0.48199 (16)	0.0171 (5)	
N2	0.63835 (17)	0.8521 (3)	0.34618 (16)	0.0192 (5)	
C1	0.83654 (19)	0.6170 (3)	0.39116 (19)	0.0169 (5)	
C2	0.8582 (2)	0.4680 (3)	0.38683 (19)	0.0183 (6)	
C3	0.8920 (2)	0.3773 (3)	0.4644 (2)	0.0198 (6)	
H3	0.9064	0.2750	0.4591	0.024*	
C4	0.9038 (2)	0.4366 (3)	0.5468 (2)	0.0198 (6)	
C5	0.8855 (2)	0.5884 (3)	0.55734 (19)	0.0180 (6)	
C6	0.85181 (19)	0.6765 (3)	0.47856 (19)	0.0160 (5)	
C7	0.8970 (2)	0.6608 (4)	0.64017 (19)	0.0208 (6)	
H7	0.9187	0.6058	0.6949	0.025*	
C8	0.8765 (2)	0.8106 (4)	0.6412 (2)	0.0230 (6)	
H8	0.8843	0.8598	0.6964	0.028*	
C9	0.8441 (2)	0.8900 (3)	0.5600 (2)	0.0202 (6)	
H9	0.8310	0.9939	0.5612	0.024*	
C10	0.6471 (2)	1.1032 (3)	0.39562 (19)	0.0183 (6)	
C11	0.6027 (2)	1.2221 (3)	0.4198 (2)	0.0217 (6)	
C12	0.5108 (2)	1.2119 (4)	0.4191 (2)	0.0248 (6)	
H12	0.4824	1.2963	0.4356	0.030*	
C13	0.4617 (2)	1.0813 (4)	0.3950 (2)	0.0250 (7)	
C14	0.5014 (2)	0.9536 (4)	0.37007 (19)	0.0218 (6)	
C15	0.5937 (2)	0.9681 (3)	0.37025 (19)	0.0185 (5)	
C16	0.4580 (2)	0.8118 (4)	0.3460 (2)	0.0288 (7)	
H16	0.3959	0.7965	0.3449	0.035*	
C17	0.5055 (2)	0.6976 (4)	0.3246 (2)	0.0299 (7)	
H17	0.4771	0.6018	0.3099	0.036*	
C18	0.5961 (2)	0.7209 (4)	0.3242 (2)	0.0251 (6)	
H18	0.6280	0.6409	0.3077	0.030*	

### Atomic displacement parameters (Å^2^)

**Table:**

	*U*^11^	*U*^22^	*U*^33^	*U*^12^	*U*^13^	*U*^23^
Sn1	0.01867 (11)	0.01519 (11)	0.01871 (11)	0.00298 (7)	0.00712 (8)	0.00097 (7)
Cl1	0.0364 (4)	0.0257 (4)	0.0232 (4)	0.0040 (3)	0.0070 (3)	0.0099 (3)
Cl2	0.0428 (4)	0.0173 (3)	0.0204 (3)	0.0031 (3)	0.0103 (3)	−0.0020 (3)
Cl3	0.0169 (3)	0.0521 (5)	0.0357 (4)	0.0022 (3)	0.0114 (3)	0.0054 (4)
Cl4	0.0308 (4)	0.0211 (4)	0.0559 (5)	−0.0008 (3)	0.0220 (4)	−0.0073 (4)
Cl5	0.0293 (4)	0.0319 (4)	0.0251 (4)	0.0071 (3)	0.0091 (3)	0.0065 (3)
Cl6	0.0314 (4)	0.0304 (4)	0.0410 (5)	−0.0019 (3)	0.0167 (4)	−0.0024 (4)
O1	0.0251 (10)	0.0166 (10)	0.0150 (9)	0.0032 (8)	0.0063 (8)	0.0006 (8)
O2	0.0167 (10)	0.0175 (10)	0.0293 (11)	−0.0003 (8)	0.0107 (9)	−0.0020 (8)
N1	0.0153 (11)	0.0178 (12)	0.0189 (11)	0.0009 (9)	0.0068 (9)	−0.0013 (9)
N2	0.0204 (12)	0.0201 (12)	0.0162 (11)	−0.0008 (10)	0.0052 (9)	0.0016 (9)
C1	0.0157 (13)	0.0172 (13)	0.0184 (13)	−0.0007 (10)	0.0066 (10)	0.0006 (10)
C2	0.0206 (14)	0.0166 (13)	0.0186 (13)	0.0006 (11)	0.0078 (11)	−0.0016 (11)
C3	0.0189 (14)	0.0174 (13)	0.0232 (14)	0.0001 (11)	0.0074 (11)	0.0014 (11)
C4	0.0168 (13)	0.0226 (15)	0.0186 (13)	−0.0022 (11)	0.0046 (11)	0.0058 (11)
C5	0.0147 (13)	0.0237 (15)	0.0153 (13)	−0.0010 (11)	0.0047 (10)	0.0021 (11)
C6	0.0132 (12)	0.0164 (13)	0.0193 (13)	−0.0006 (10)	0.0069 (10)	−0.0005 (10)
C7	0.0182 (13)	0.0281 (16)	0.0167 (13)	−0.0008 (12)	0.0067 (11)	0.0017 (12)
C8	0.0203 (14)	0.0306 (17)	0.0191 (14)	−0.0005 (12)	0.0083 (11)	−0.0041 (12)
C9	0.0178 (13)	0.0207 (14)	0.0234 (14)	−0.0012 (11)	0.0089 (11)	−0.0046 (11)
C10	0.0181 (13)	0.0205 (14)	0.0173 (13)	0.0009 (11)	0.0074 (11)	0.0025 (11)
C11	0.0217 (14)	0.0201 (14)	0.0245 (14)	0.0027 (12)	0.0094 (12)	0.0016 (11)
C12	0.0228 (15)	0.0299 (17)	0.0233 (15)	0.0079 (13)	0.0099 (12)	0.0042 (12)
C13	0.0146 (13)	0.0381 (18)	0.0224 (15)	0.0029 (12)	0.0067 (11)	0.0061 (13)
C14	0.0189 (14)	0.0290 (16)	0.0159 (13)	−0.0013 (12)	0.0041 (11)	0.0039 (12)
C15	0.0176 (13)	0.0221 (14)	0.0157 (12)	0.0004 (11)	0.0056 (10)	0.0034 (11)
C16	0.0214 (15)	0.041 (2)	0.0229 (15)	−0.0091 (14)	0.0058 (12)	0.0010 (14)
C17	0.0296 (17)	0.0285 (17)	0.0276 (16)	−0.0131 (14)	0.0052 (13)	−0.0049 (13)
C18	0.0288 (16)	0.0243 (16)	0.0208 (14)	−0.0022 (13)	0.0070 (12)	−0.0010 (12)

### Geometric parameters (Å, °)

**Table:**

Sn1—O1	2.055 (2)	C4—C5	1.405 (4)
Sn1—O2	2.061 (2)	C5—C6	1.413 (4)
Sn1—N1	2.222 (2)	C5—C7	1.419 (4)
Sn1—N2	2.244 (2)	C7—C8	1.374 (5)
Sn1—Cl6	2.3122 (9)	C7—H7	0.9500
Sn1—Cl5	2.3561 (8)	C8—C9	1.400 (4)
Cl1—C4	1.737 (3)	C8—H8	0.9500
Cl2—C2	1.726 (3)	C9—H9	0.9500
Cl3—C13	1.735 (3)	C10—C11	1.383 (4)
Cl4—C11	1.726 (3)	C10—C15	1.432 (4)
O1—C1	1.332 (3)	C11—C12	1.401 (4)
O2—C10	1.321 (4)	C12—C13	1.366 (5)
N1—C9	1.316 (4)	C12—H12	0.9500
N1—C6	1.369 (4)	C13—C14	1.411 (5)
N2—C18	1.323 (4)	C14—C15	1.412 (4)
N2—C15	1.365 (4)	C14—C16	1.418 (5)
C1—C2	1.378 (4)	C16—C17	1.361 (5)
C1—C6	1.425 (4)	C16—H16	0.9500
C2—C3	1.411 (4)	C17—C18	1.400 (5)
C3—C4	1.362 (4)	C17—H17	0.9500
C3—H3	0.9500	C18—H18	0.9500
			
O1—Sn1—O2	160.55 (8)	N1—C6—C1	116.0 (3)
O1—Sn1—N1	77.91 (8)	C5—C6—C1	122.5 (3)
O2—Sn1—N1	88.76 (9)	C8—C7—C5	119.9 (3)
O1—Sn1—N2	87.73 (9)	C8—C7—H7	120.0
O2—Sn1—N2	76.73 (9)	C5—C7—H7	120.0
N1—Sn1—N2	84.03 (9)	C7—C8—C9	119.4 (3)
O1—Sn1—Cl6	98.77 (6)	C7—C8—H8	120.3
O2—Sn1—Cl6	95.19 (6)	C9—C8—H8	120.3
N1—Sn1—Cl6	89.56 (7)	N1—C9—C8	122.0 (3)
N2—Sn1—Cl6	169.75 (7)	N1—C9—H9	119.0
O1—Sn1—Cl5	93.79 (6)	C8—C9—H9	119.0
O2—Sn1—Cl5	97.26 (6)	O2—C10—C11	124.0 (3)
N1—Sn1—Cl5	168.73 (7)	O2—C10—C15	120.0 (3)
N2—Sn1—Cl5	88.07 (6)	C11—C10—C15	116.0 (3)
Cl6—Sn1—Cl5	99.32 (3)	C10—C11—C12	122.2 (3)
C1—O1—Sn1	115.39 (17)	C10—C11—Cl4	119.5 (2)
C10—O2—Sn1	116.24 (18)	C12—C11—Cl4	118.3 (2)
C9—N1—C6	120.2 (3)	C13—C12—C11	120.4 (3)
C9—N1—Sn1	129.2 (2)	C13—C12—H12	119.8
C6—N1—Sn1	110.64 (18)	C11—C12—H12	119.8
C18—N2—C15	120.1 (3)	C12—C13—C14	121.5 (3)
C18—N2—Sn1	128.8 (2)	C12—C13—Cl3	119.0 (3)
C15—N2—Sn1	111.08 (19)	C14—C13—Cl3	119.5 (3)
O1—C1—C2	123.4 (3)	C15—C14—C13	116.7 (3)
O1—C1—C6	120.0 (3)	C15—C14—C16	117.0 (3)
C2—C1—C6	116.6 (3)	C13—C14—C16	126.3 (3)
C1—C2—C3	122.1 (3)	N2—C15—C14	121.6 (3)
C1—C2—Cl2	119.5 (2)	N2—C15—C10	115.3 (3)
C3—C2—Cl2	118.4 (2)	C14—C15—C10	123.2 (3)
C4—C3—C2	120.0 (3)	C17—C16—C14	119.8 (3)
C4—C3—H3	120.0	C17—C16—H16	120.1
C2—C3—H3	120.0	C14—C16—H16	120.1
C3—C4—C5	121.5 (3)	C16—C17—C18	120.1 (3)
C3—C4—Cl1	119.1 (2)	C16—C17—H17	119.9
C5—C4—Cl1	119.4 (2)	C18—C17—H17	119.9
C4—C5—C6	117.2 (3)	N2—C18—C17	121.4 (3)
C4—C5—C7	125.7 (3)	N2—C18—H18	119.3
C6—C5—C7	117.0 (3)	C17—C18—H18	119.3
N1—C6—C5	121.5 (3)		
			
O2—Sn1—O1—C1	−49.4 (3)	Sn1—N1—C6—C1	0.3 (3)
N1—Sn1—O1—C1	−1.69 (19)	C4—C5—C6—N1	179.5 (3)
N2—Sn1—O1—C1	−86.08 (19)	C7—C5—C6—N1	0.1 (4)
Cl6—Sn1—O1—C1	85.96 (19)	C4—C5—C6—C1	−0.1 (4)
Cl5—Sn1—O1—C1	−173.99 (18)	C7—C5—C6—C1	−179.5 (3)
O1—Sn1—O2—C10	−45.4 (4)	O1—C1—C6—N1	−1.9 (4)
N1—Sn1—O2—C10	−91.7 (2)	C2—C1—C6—N1	179.0 (2)
N2—Sn1—O2—C10	−7.57 (19)	O1—C1—C6—C5	177.8 (3)
Cl6—Sn1—O2—C10	178.82 (19)	C2—C1—C6—C5	−1.3 (4)
Cl5—Sn1—O2—C10	78.7 (2)	C4—C5—C7—C8	179.9 (3)
O1—Sn1—N1—C9	−179.7 (3)	C6—C5—C7—C8	−0.7 (4)
O2—Sn1—N1—C9	−13.9 (2)	C5—C7—C8—C9	0.2 (4)
N2—Sn1—N1—C9	−90.7 (3)	C6—N1—C9—C8	−1.5 (4)
Cl6—Sn1—N1—C9	81.3 (2)	Sn1—N1—C9—C8	178.9 (2)
Cl5—Sn1—N1—C9	−136.4 (3)	C7—C8—C9—N1	0.9 (5)
O1—Sn1—N1—C6	0.71 (18)	Sn1—O2—C10—C11	−173.6 (2)
O2—Sn1—N1—C6	166.44 (19)	Sn1—O2—C10—C15	7.9 (3)
N2—Sn1—N1—C6	89.65 (19)	O2—C10—C11—C12	−178.9 (3)
Cl6—Sn1—N1—C6	−98.36 (18)	C15—C10—C11—C12	−0.3 (4)
Cl5—Sn1—N1—C6	43.9 (4)	O2—C10—C11—Cl4	0.0 (4)
O1—Sn1—N2—C18	−8.2 (3)	C15—C10—C11—Cl4	178.6 (2)
O2—Sn1—N2—C18	−176.5 (3)	C10—C11—C12—C13	0.6 (5)
N1—Sn1—N2—C18	−86.3 (3)	Cl4—C11—C12—C13	−178.3 (2)
Cl6—Sn1—N2—C18	−137.9 (3)	C11—C12—C13—C14	−0.1 (5)
Cl5—Sn1—N2—C18	85.6 (3)	C11—C12—C13—Cl3	179.6 (2)
O1—Sn1—N2—C15	174.68 (19)	C12—C13—C14—C15	−0.7 (4)
O2—Sn1—N2—C15	6.47 (18)	Cl3—C13—C14—C15	179.6 (2)
N1—Sn1—N2—C15	96.60 (19)	C12—C13—C14—C16	178.4 (3)
Cl6—Sn1—N2—C15	45.0 (5)	Cl3—C13—C14—C16	−1.3 (4)
Cl5—Sn1—N2—C15	−91.45 (18)	C18—N2—C15—C14	−1.8 (4)
Sn1—O1—C1—C2	−178.4 (2)	Sn1—N2—C15—C14	175.6 (2)
Sn1—O1—C1—C6	2.5 (3)	C18—N2—C15—C10	178.0 (3)
O1—C1—C2—C3	−177.7 (3)	Sn1—N2—C15—C10	−4.6 (3)
C6—C1—C2—C3	1.3 (4)	C13—C14—C15—N2	−179.2 (3)
O1—C1—C2—Cl2	0.8 (4)	C16—C14—C15—N2	1.6 (4)
C6—C1—C2—Cl2	179.9 (2)	C13—C14—C15—C10	1.1 (4)
C1—C2—C3—C4	0.1 (4)	C16—C14—C15—C10	−178.1 (3)
Cl2—C2—C3—C4	−178.4 (2)	O2—C10—C15—N2	−1.7 (4)
C2—C3—C4—C5	−1.7 (4)	C11—C10—C15—N2	179.7 (3)
C2—C3—C4—Cl1	176.8 (2)	O2—C10—C15—C14	178.1 (3)
C3—C4—C5—C6	1.6 (4)	C11—C10—C15—C14	−0.6 (4)
Cl1—C4—C5—C6	−176.9 (2)	C15—C14—C16—C17	0.1 (4)
C3—C4—C5—C7	−179.0 (3)	C13—C14—C16—C17	−179.1 (3)
Cl1—C4—C5—C7	2.5 (4)	C14—C16—C17—C18	−1.5 (5)
C9—N1—C6—C5	1.0 (4)	C15—N2—C18—C17	0.2 (4)
Sn1—N1—C6—C5	−179.3 (2)	Sn1—N2—C18—C17	−176.6 (2)
C9—N1—C6—C1	−179.4 (3)	C16—C17—C18—N2	1.5 (5)
